# Prebiotic Effects of Partially Hydrolyzed Guar Gum on the Composition and Function of the Human Microbiota—Results from the PAGODA Trial

**DOI:** 10.3390/nu12051257

**Published:** 2020-04-28

**Authors:** Simon J. Reider, Simon Moosmang, Judith Tragust, Lovro Trgovec-Greif, Simon Tragust, Lorenz Perschy, Nicole Przysiecki, Sonja Sturm, Herbert Tilg, Hermann Stuppner, Thomas Rattei, Alexander R. Moschen

**Affiliations:** 1 Christian Doppler Laboratory for Mucosal Immunology, Medical University Innsbruck, 6020 Innsbruck, Austria; simon.reider@i-med.ac.at (S.J.R.); Judith.Tragust@hotmail.de (J.T.); Nicole.Przysiecki@i-med.ac.at (N.P.); 2 Department of Internal Medicine I, Gastroenterology, Hepatology, Endocrinology & Metabolism, Medical University Innsbruck, 6020 Innsbruck, Austria; herbert.tilg@i-med.ac.at; 3 Institute of Pharmacy/Pharmacognosy & Center for Molecular Biosciences Innsbruck, Leopold-Franzens Universität, 6020 Innsbruck, Austria; Simon-Moosmang@web.de (S.M.); sonja.sturm@uibk.ac.at (S.S.); hermann.stuppner@i-med.ac.at (H.S.); 4 Division of Computational Systems Biology, Department of Microbiology, University of Vienna, 1010 Vienna, Austria; lovro.lovro@hotmail.com (L.T.-G.); lorenz.perschy@icloud.com (L.P.); thomas.rattei@univie.ac.at (T.R.); 5 General Zoology Institute of Biology, University Halle, 06108 Halle, Germany; simon.tragust@zoologie.uni-halle.de

**Keywords:** prebiotics, fiber, partially hydrolyzed guar gum, PHGG, microbiota, short-chain fatty acids, SCFA

## Abstract

(1) Background: Alterations in the structural composition of the human gut microbiota have been identified in various disease entities along with exciting mechanistic clues by reductionist gnotobiotic modeling. Improving health by beneficially modulating an altered microbiota is a promising treatment approach. Prebiotics, substrates selectively used by host microorganisms conferring a health benefit, are broadly used for dietary and clinical interventions. Herein, we sought to investigate the microbiota-modelling effects of the soluble fiber, partially hydrolyzed guar gum (PHGG). (2) Methods: We performed a 9 week clinical trial in 20 healthy volunteers that included three weeks of a lead-in period, followed by three weeks of an intervention phase, wherein study subjects received 5 g PHGG up to three times per day, and concluding with a three-week washout period. A stool diary was kept on a daily basis, and clinical data along with serum/plasma and stool samples were collected on a weekly basis. PHGG-induced alterations of the gut microbiota were studied by 16S metagenomics of the V1–V3 and V3–V4 regions. To gain functional insight, we further studied stool metabolites using nuclear magnetic resonance (NMR) spectroscopy. (3) Results: In healthy subjects, PHGG had significant effects on stool frequency and consistency. These effects were paralleled by changes in α- (species evenness) and β-diversity (Bray–Curtis distances), along with increasing abundances of metabolites including butyrate, acetate and various amino acids. On a taxonomic level, PHGG intake was associated with a bloom in *Ruminococcus*, *Fusicatenibacter*, *Faecalibacterium* and *Bacteroides* and a reduction in *Roseburia*, *Lachnospiracea* and *Blautia*. The majority of effects disappeared after stopping the prebiotic and most effects tended to be more pronounced in male participants. (4) Conclusions: Herein, we describe novel aspects of the prebiotic PHGG on compositional and functional properties of the healthy human microbiota.

## 1. Introduction

The human intestinal tract is populated by a complex microbial community, providing essential metabolic and immunological functions to the host, influencing normal physiology as well as disease susceptibility [1]. Using metagenomic techniques, more than 2000 bacterial species have been detected in the intestinal tract and approximately 100–1000 different bacterial taxa are present in a given individual [2,3]. The majority of these bacteria are members of the phyla Bacteroidetes and Firmicutes, while others belong to Proteobacteria, Actinobacteria and Verrucomicrobia [4,5]. The composition of the microbiota in the steady state is characterized by high diversity and marked inter-individual variability. On a functional level, however, different species share metabolic properties, resulting in set of core metabolic functions that are shared by the healthy microbiota. Bacterial communities are stabilized by microbe–microbe interactions and exhibit a remarkable resilience to perturbations. In spite of these mechanisms, alterations of the microbiota have been observed in numerous pathologic conditions [6,7,8,9,10,11].

Accordingly, there is considerable interest in approaches aimed at altering microbial composition and functions counteracting disease-driving factors and promoting health-beneficial properties. Various approaches including dietary and non-dietary interventions have been described and probiotic and prebiotic modalities are most widely used in clinical practice. Following the definition of probiotics, ‘live microorganisms which when administered in adequate amounts confer a health benefit’ [12], a prebiotic is considered ‘a substrate that is selectively utilized by host microorganisms conferring a health benefit’ [13]. These features are thought to demarcate a prebiotic from dietary fibers. Accordingly, even if dietary fibers such as pectins [14] and xylans [15] may cause alterations of gut commensal compositions, either the criterion of selective utilization or a health benefit have not yet been sufficiently demonstrated [13].

Partially hydrolyzed guar gum (PHGG) is a soluble dietary fiber with a linear backbone of β-1,4-linked D-mannose residues, with α-D-galactose residues 1,6-linked to every second mannose. This galactomannan completely dissolves in water, does not form a gel and demonstrates prebiotic properties as previously defined by increasing the abundance of Lactobacilli and Bifidobacteria as well as colonic SCFA contents [16,17]. In functional gastrointestinal disease such as irritable bowel syndrome (IBS), PHGG showed efficacy in improving bloating [18] and improving IBS-related symptoms and quality of life [19] compared to placebo. Fermentation of PHGG by the microbiota increases the abundance of short-chain fatty acids (SCFAs) including acetate, propionate and butyrate. SCFAs are an important energy source for colonic epithelial cells and have a variety of regulatory functions on gut physiology, metabolism and immunity [20].

Despite some evidence for its efficacy and its clinical and nutritional use [21], the prebiotic effects of PHGG, in light of the prebiotic definition and methodological progresses that have been made in studying the microbiota in recent years, are inadequately defined. The aim of this study was to decipher and define the overall prebiotic properties of PHGG in healthy volunteers focusing on taxonomic and functional (i.e., metabolites) properties using state-of-the-art methodologies.

## 2. Materials and Methods

### 2.1. Study Design and Criteria for Participation

**Figure 1:**
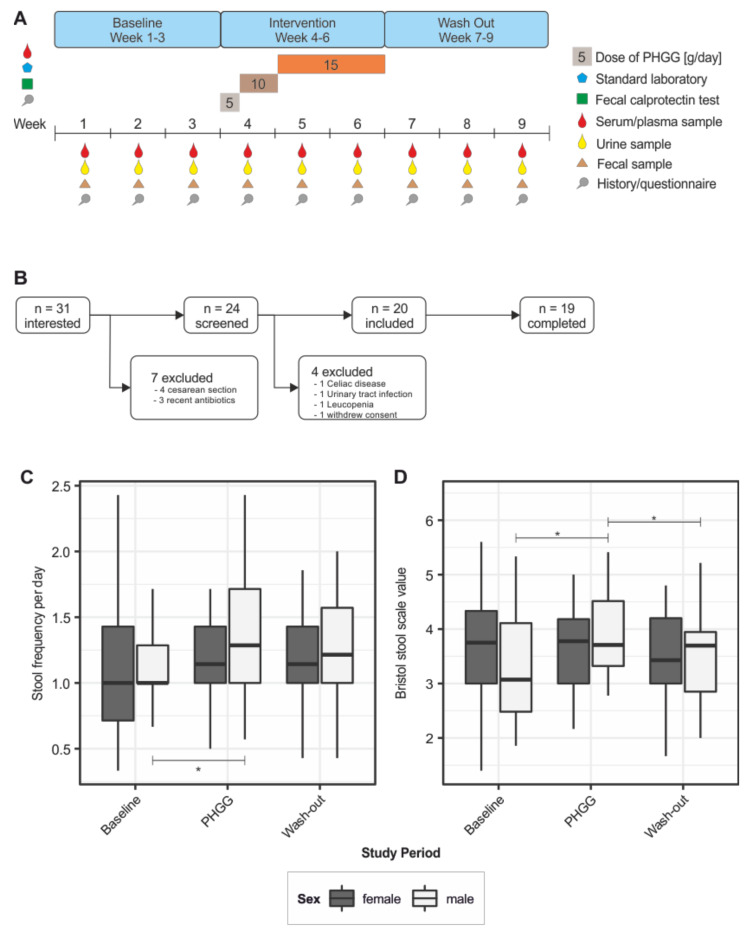
Study design and clinical effects of PHGG supplementation. (**A**) Outline of the design and sampling procedure of the PAGODA study; (**B**) screening process and number of participants included in this study; (**C**) effect of PHGG supplementation on stool frequency; (**D**) effect of PHGG supplementation on stool consistency according to the Bristol stool scale value. PHGG = partially hydrolyzed guar gum; * *p* < 0.05; linear mixed model.

The PAGODA study is a longitudinal trial including 20 healthy volunteers undergoing supplementation with PHGG. PHGG was from one specific lot and kindly provided by Nestlé Austria. The trial was performed with approval from the ethics committee of the Medical University Innsbruck (Number: AN2016-0244) and informed consent was obtained from all study subjects. The overall duration of this study was nine weeks, split in three periods (see Figure 1A): during the first three weeks, baseline values were obtained; in weeks 4–6, PHGG was administered in increasing doses (5 g, 10 g, and 15 g per day); finally, PHGG supplementation was stopped and the participants were monitored for another three weeks to distinguish transient from persistent changes. At the baseline, a medical history and information on dietary habits were obtained. Participants were advised to maintain their baseline dietary habits throughout this study. Furthermore, laboratory chemistry analyses were performed during screening in order to exclude candidates with abnormal test values (blood count, C-reactive protein, fecal calprotectin). During this study, sampling was performed once a week. The sample set included fecal matter, urine, serum and plasma. Fecal samples were collected in a Sarstedt stool container within 12 h before and kept at 4 °C until the weekly study visit. Samples were immediately aliquoted and stored at −80 °C until further workup. Additionally, clinical data and information on current well-being were collected on a weekly basis using questionnaires. To be eligible for study participation, the following criteria had to be met: no history of antibiotic therapy within 3 months before this study, history of vaginal delivery and having been breast fed for at least 3 months. Participants were excluded from this study in the case of an acute or chronic gastrointestinal disease, history of major abdominal surgery (appendectomy excluded), if they consumed dietary supplements or probiotics, were on an exclusive vegetarian or vegan diet or showed abnormal laboratory test values at baseline.

### 2.2. The 16.S Amplicon-Based Metagenomics

During this study, fecal samples were collected weekly by the participants. Microbial DNA was isolated from these samples using the Qiagen PowerSoil Kit according to the manufacturer’s instructions. Isolated DNA was shipped to IMGM (Munich, Germany) for sequencing on the Illumina MISEQ platform using primers targeting the V1–V3 and V3–V4 regions of the bacterial 16S gene [22] (primer sequences detailed in Table S1) aiming for 100,000 reads per region. Generated 16S sequence data are available from the European Nucleotide Archive (accession number ERP120904). All scripts related to our analyses will be provided by the authors upon reasonable request (to SJR). The sequencing results were checked using FASTQC [23] and processed in mothur according to a published protocol (version 1.40.5) [24,25]. Briefly, reads were filtered for a minimum length of 275 nucleotides in prinseq [26]. Filtered fasta files were merged and aligned to the SILVA v128 database [27]. Chimeric reads were removed using vsearch [28]. The SILVA v128 database was also used for taxonomic classification. Finally, operational taxonomic unit (OTU) clustering was performed at a level of 97% identity separately for the V1–V3 and the V3–V4 data. This resulted in 7,509,559 and 7,855,428 high-quality reads clustering into 2,157,950 and 825,683 OTUs in the V1–V3 and the V3–V4 dataset, respectively. After excluding OTUs present only once in the dataset (singleton OTUs), 231,695 (V1–V3) and 177,260 OTUs (V3–V4) were retained.

### 2.3. NMR Spectroscopy

For nuclear magnetic resonance (NMR) spectroscopy, watery extracts from fecal samples were generated by ultrasonication and repeated freeze–thaw cycles. NMR analysis was performed on a Bruker Avance II 600 MHz. A standard Bruker noesypr1d sequence was used to suppress signals from water molecules. Acquisition parameters for the spectra were 64 scans, a spectral width of 7288 Hz collected into 32K data points, an acquisition time of 2.24 s, and an inter-scan relaxation delay of 5 s. The free induction decay (FID) obtained was multiplied by 0.3 Hz of exponential line broadening before Fourier transformation. The spectra were referenced to TSP (chemical shift 0 ppm), phased, and baseline corrected in Topspin 3.0 software (Bruker, Billerica, MA, USA). Relative quantification of selected 1H resonance was performed by integration of peak areas using Topspin 3.0 (Bruker) [29]. NMR spectra were imported to Matlab R2010b (The Mathworks, Natick, MA, USA), and the misalignments of the spectra were corrected using the icoshift algorithm, based on the correlational shifting of spectral intervals [30]. The spectrum with the highest correlation to the rest of the spectra in the matrix was used as a reference. The 9.5 to 12 ppm region and the region containing residual water resonance (4.8. to 4.7 ppm) were removed from the aligned spectra. The spectra were normalized to unit area and pareto scaled before principal component analysis (PCA) using the PLS Toolbox in MATLAB R2010b. Alternatively, metabolites were identified using automatic statistical identification in complex spectra (ASICS). This approach is based on a library of NMR spectra containing information on 191 pure metabolites [31]. Alternatively, manual attribution of peak identities was performed based on shift values from reference spectra obtained from measurements of pure substances and spike in of pure substances in fecal extracts.

### 2.4. Statistics

Clinical data were analyzed in R using a linear mixed model (study period, sex, interaction of study period and sex as fixed effect, study week and subject as random effect). Metagenomic data were imported into phyloseq [32] in R and analyzed according to established procedures. Within-sample (α) diversity was calculated from unfiltered samples; for all other calculations, singleton OTUs were removed from the database. Bray–Curtis dissimilarity was calculated on a rarefied dataset and primary coordinate analysis plots were generated. Samples with less than 1000 reads were removed from the database. Vegan [33] was used to perform permutational analysis of variance (PERMANOVA) with study week and subject as predictive variables. Testing for differentially abundant OTUs was performed using DESeq2 using all OTUs, with an absolute abundance of at least three in at least 10% of samples [34,35]. Differential abundance was assumed only if the log2 fold change exceeded 1.7.

NMR spectra were analyzed in a targeted approach integrating intensities of known peaks corresponding to SCFAs, galactose, mannose and PHGG. These peak identities were confirmed by additional NMR measurements of pure substances in the same solvent. Additionally, peak intensities were correlated to microbial abundances in a blinded fashion using SparCC with default parameters [36]. The list of correlations was filtered for interactions with a *p* value of < 0.05 and a correlation coefficient of >0.2. The peaks included in this filtered correlation matrix were then annotated by hand as described and the network was drawn in Cytoscape v3.7.1 [37].

## 3. Results

### 3.1. Study Cohort and Baseline Nutritional Characteristics

Thirty-one individuals were interested in participating in this study. After controlling for inclusion/exclusion criteria, 24 underwent screening. Three individuals had to be excluded due to pre-existing medical conditions discovered during screening (celiac disease, urinary tract infection and leucopenia) and one individual declined to participate. Twenty volunteers were included and 19 of them completed this study (8 males, 11 females; Figure 1B). The mean age of study participants was 27.8 years (minimum 20, maximum 46 years) and the mean BMI was 21.7 ± 2.4. Laboratory values and pre-existing medical conditions that did not preclude study participation are listed in Tables S2 and S3. Baseline nutritional habits were characterized in detail using a questionnaire as previously described [38]. Both male and female participants predominantly had three meals a day, with no significance difference in the intake of carbohydrates or dairy products. However, female participants reported higher intake of fruit and fiber, and reduced intake of meat (Figure S1).

### 3.2. Effect of PHGG Intake on Intestinal Well-Being and Bowel Function

First, specific clinical effects of PHGG supplementation on intestinal well-being and bowel function were studied based on the weekly questionnaires. A linear model showed significant changes in the number of bowel movements per day (*p* < 0.01), which was particularly attributable to the male subpopulation (*p* < 0.05). PHGG intake was associated with a significant increase in the number of daily stools (1.13 ± 0.46 vs. 1.27 ± 0.47, *p* < 0.01). This effect did not persist after the end of the intervention period (washout period: 1.22 ± 0.41). These differences were gender dependent: While there was an increase in stool frequency and consistency (BSS value) in male participants, female participants did not experience such effects upon PHGG supplementation (Figure 1C,D). Table S3 summarizes results of the intestinal well-being and symptoms questionnaire during the 9 week study period.

### 3.3. PHGG-Associated Changes of Microbial Community Structures

**Figure 2:**
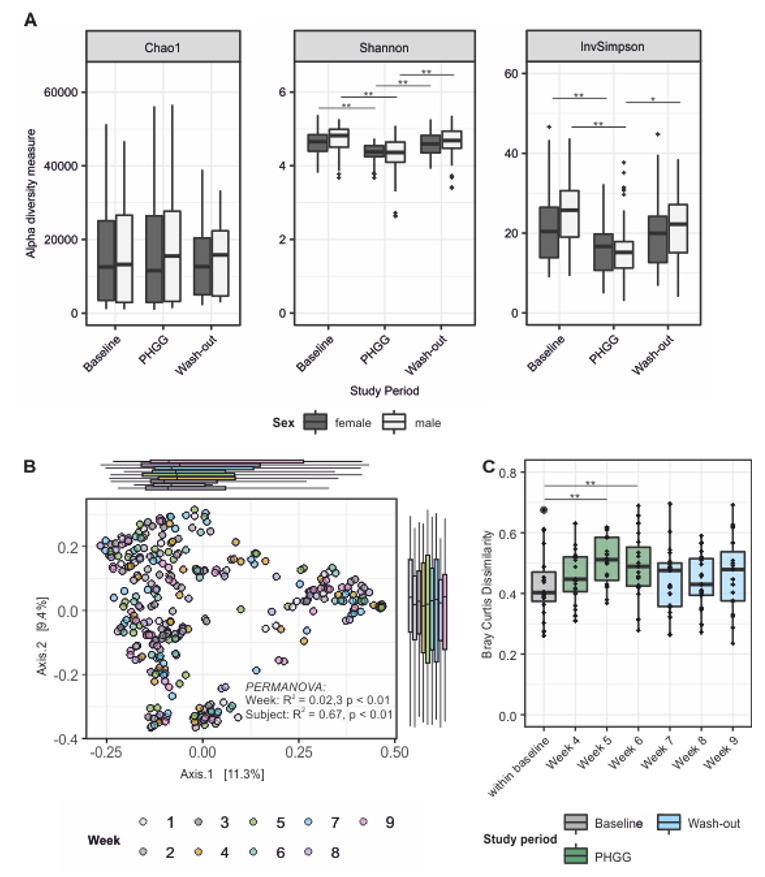
Impact of PHGG supplementation on intestinal microbiota composition. (**A**) α-diversity decreases during intervention in both sexes and returns to baseline during the washout period (pairwise Wilcoxon Test). (**B**) Principal component analysis of microbial compositions over time; study week (i.e., PHGG supplementation status) as determinant was significant (PERMANOVA permutational analysis of variance). (**C**) Pairwise analyses of Bray–Curtis dissimilarities over time (Wilcoxon test). PHGG = partially hydrolyzed guar gum; * *p* < 0.05, ** *p* < 0.01.

Samples from all three study periods were compared regarding α-diversity (Figure 2A and Figure S2A). While the number of observed species remained unchanged during PHGG supplementation (species richness, the Chao1 index), species evenness (the Shannon and inverse Simpson indices) decreased significantly (*p* < 0.05; Kruskal–Wallis test). All measures of α-diversity returned to baseline values during the washout period. These effects reached statistical significance in both sexes in the V3–V4 dataset (Figure 2A), but only in the V1–V3 region in males (Figure S2A). No differences between male and female participants were found for each study period (*p* > 0.05, Bonferroni corrected pairwise Wilcoxon tests). Bray–Curtis dissimilarity was used to calculate β-diversity (Figure 2B and Figure S2B). PHGG supplementation significantly affected microbial community composition (R^2^ = 0.023 and 0.020 respectively; *p* < 0.01; permutational analysis of variance). This resulted in a significant difference of pairwise Bray–Curtis dissimilarities over time between the baseline and the final week of the intervention period (week 6; *p* < 0.05, Wilcoxon test). After stopping PHGG intervention, paired Bray–Curtis dissimilarities quickly returned to individuals’ baseline variability measured between weeks 1 and 3. At a personalized level, the reconfiguration of the microbial composition was highly individual (Figures S3 and S4). Inter-individual differences in microbial community composition accounted for most of the variance (factor subject; R2 = 0.67 and 0.68, respectively; *p* < 0.01, permutational analysis of variance).

**Figure 3:**
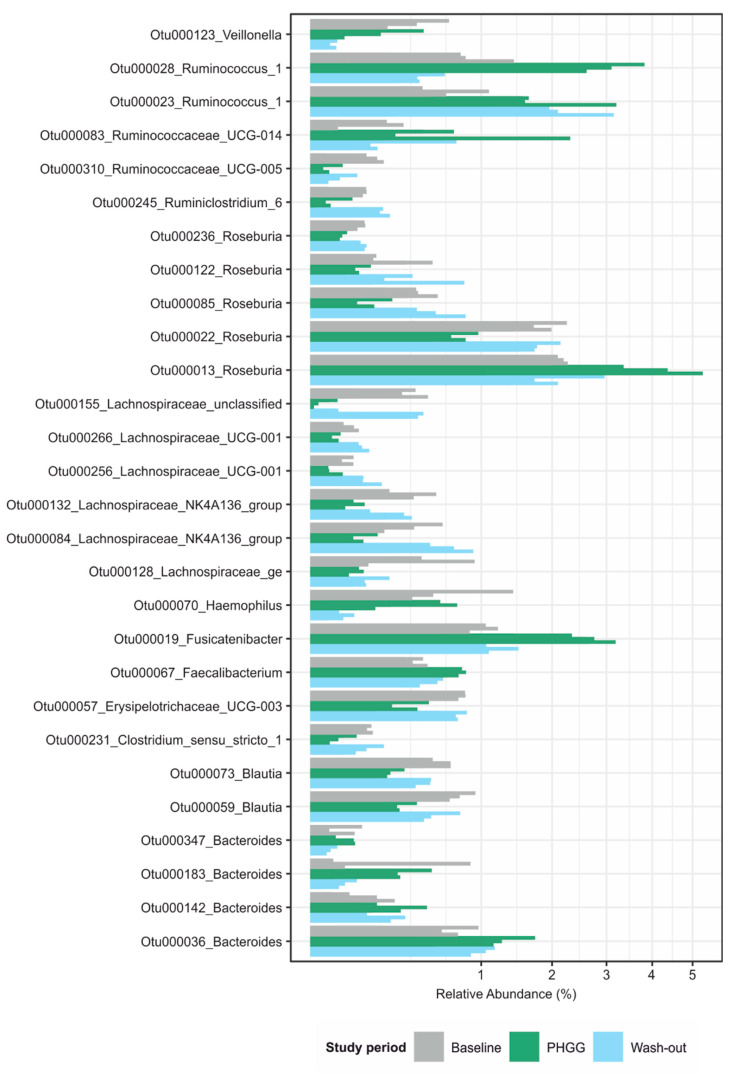
Significantly differentially abundant OTUs and their mapping on the genus level in the V3–V4 dataset. Testing was carried out using a negative binomial model implemented in DESeq2 and a cutoff of 0.01 for the adjusted *p*-value. OTU = operational taxonomic unit; UCG = unknown classification group; PHGG = partially hydrolyzed guar gum.

To identify potential causes underlying the observed changes in diversity measures, we proceeded to test for differentially abundant bacterial taxa between study periods, accounting for subject as an additional factor (false discovery rate-adjusted *p* < 0.01, negative binomial Wald’s test). Using the V3–V4 dataset (Figure 3), 28 taxa predominantly from the Firmicutes phylum were detected. In most of these taxa, an observed change during the intervention period was not sustained during the washout period, (i.e., taxon abundance quickly reverted to baseline values). PHGG had suppressing as well as enhancing effects on the abundance of specific OTUs. Notably, a few OTUs exhibited persistent changes. These belonged to the *Ruminococcus*, *Faecalibacterium* and *Bacteroides* genus. In comparison, in the V1–V3 dataset (Figure S5) 14 OTUs were significantly differentially abundant. Again, most of these OTUs were only transiently affected but one OTU corresponding to Faecalibacterium remained increased during the washout period. These findings widely overlapped between both sequenced 16S regions. OTUs whose abundance was negatively associated with PHGG supplementation mapped to the genera *Roseburia*, *Lachnospiracea* and *Blautia*, while those that bloomed during the intervention period belonged to *Ruminococcus*, *Fusicatenibacter*, *Faecalibacterium* and *Bacteroides* (Figure 3 and Figure S5).

### 3.4. Abundance of Specific Taxa in the Steady State Correlates with Clinical Response to PHGG

**Figure 4:**
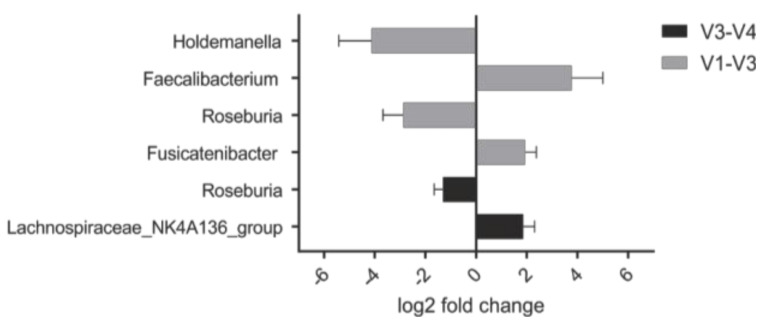
The abundance of certain taxa at baseline is higher in those that experience a clinical response to PHGG. These taxa almost exclusively belong to the order of Clostridiales. Notably, *Roseburia* is detected in both datasets. Negative binomial Wald-test comparing two groups defined by an increase in stool frequency per day larger or smaller than the median increase in this study population; all, *p* < 0.05.

As there is considerable interest in factors underlying the efficacy of a prebiotic treatment in a given individual, we aimed to identify characteristics of the microbiota during the steady state that would correlate with clinical response to PHGG. Using data from the clinical symptoms questionnaires, responders to PHGG were defined by an increase in stool frequency per day higher than the mean increase observed in the overall study population. Accordingly, future responders to PHGG intervention exhibited a higher baseline abundance of certain members of the Clostridiales order such as *Faecalibacterium*, *Fusicatenibacter* and *Roseburia* (*p* < 0.05; Figure 4).

### 3.5. PHGG-Associated Changes in the Fecal Metabolome

**Figure 5:**
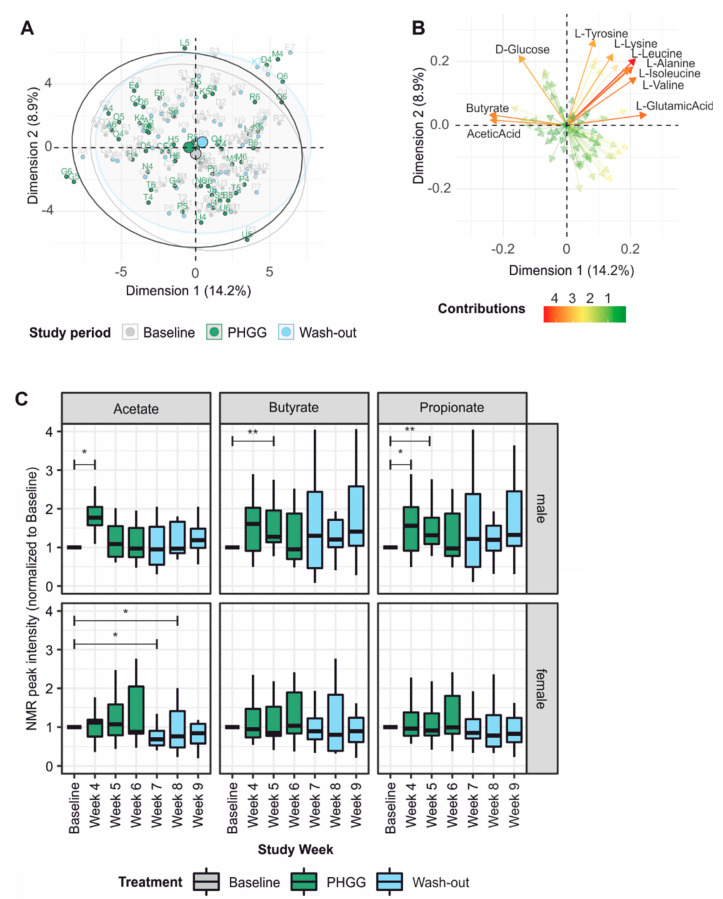
NMR spectroscopy of fecal samples: Peaks were annotated automatically (**A**,**B**) or manually using reference spectra (**C**). (**A**) Principle component analysis of metabolite profiles by study period indicates a small shift during the PHGG intervention. (**B**) Butyrate and acetate contribute most to the changes seen in **A**. (**C**) Targeted analysis of SCFA levels in fecal samples reveals sex-dependent changes over time, with most significant effects at the beginning of the intervention period. Pairwise Wilcoxon tests with Bonferroni correction.

In an untargeted approach, NMR peaks were attributed automatically using ASICS and its reference library of 191 pure NMR spectra. Principle component analysis revealed a slight shift, mainly along the first component during the PHGG intervention period (Figure 5A) and mostly due to differences in the short-chain fatty acids butyrate and acetate (Figure 5B). Various amino acids also contributed to differences between the study periods. Building upon these findings, we next annotated NMR spectra manually based upon reference spectra for the short-chain fatty acids (SCFAs) acetate, butyrate and propionate. Peak intensities were expressed as fold change compared to the individual participant’s baseline. This analysis showed sex-dependent differences in SCFA dynamics, with male participants in general experiencing larger and earlier changes during the intervention period (Figure 5C). There was a tendency towards lower butyrate levels at baseline in prospective male responders to PHGG, although this finding did not reach statistical significance (data not shown). We also attempted to measure PHGG and its metabolites galactose and mannose in the feces by comparing participants’ NMR spectra during the intervention period to spectra obtained from PHGG, galactose and mannose alone. No increase in the PHGG signal could be detected during supplementation indicating complete metabolization of the soluble fiber. Fittingly, we observed a trend towards increased fecal concentrations of galactose and mannose, the cleavage products of PHGG (Figure S6).

### 3.6. Correlation Network

**Figure 6:**
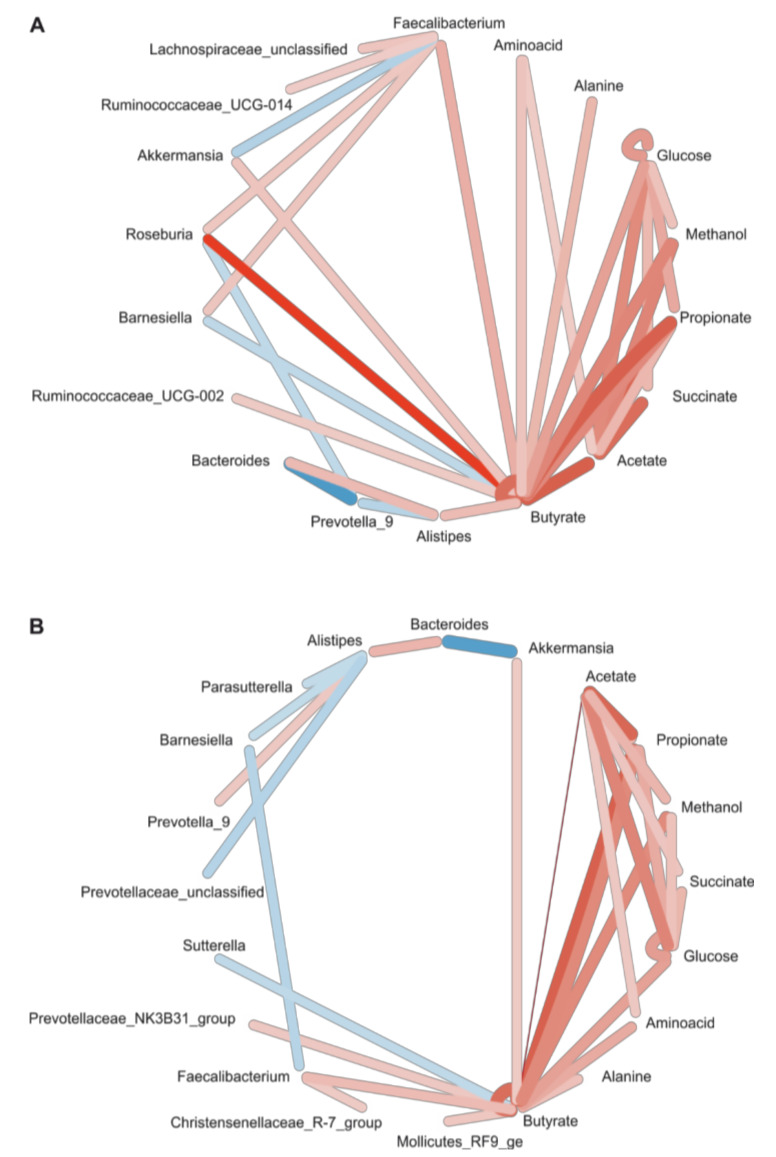
Correlation network of taxa and metabolites. Correlations between taxon abundance and peak intensities were calculated using SparCC and taxonomic abundance data (**A**) for the V3–V4 and (**B**) the V1–V3 region. Both regions reveal an interaction network centered on butyrate, with strong links between the SCFA as well as to certain taxa. In both datasets, Faecalibacterium was positively correlated with butyrate. Edge color = direction of interaction: red = positive; blue = negative; Edge weight = strength of interaction, i.e., correlation coefficient; only interactions with a two-sided *p*-value of >0.05 were included.

Fecal metabolites reflect the biochemical activity of the intestinal microbiota. Hence, we statistically assessed co-occurrence and co-exclusion of bacterial genera of the microbiota and peaks of NMR spectra, resulting in an interaction network centered on butyrate. There were strong links to other SCFAs (acetate, propionate) and certain bacterial genera. These bacteria in part overlapped with those identified by DESeq2 analysis. Bacteria of the Firmicutes phylum, e.g., certain groups of Ruminococcaceae and Lachnospiraceae as well as *Akkermansia* were positively correlated with butyrate abundance. There was a negative interaction of butyrate and *Barnesiella*, and strong negative interactions between *Bacteroides*, *Prevotella* and *Roseburia*, respectively. Importantly, the findings from the V3–V4 and V1–V3 datasets were highly comparable (Figure 6).

## 4. Discussion

Microbiota research provides us with fascinating insights into how an altered microbiota may be linked to human disease [39]. Prebiotics look back upon a long tradition in clinical nutrition and their microbiota-modulating properties along with their favorable safety fertilize a growing prebiotic market [40]. This prompted the International Scientific Association for Probiotics and Prebiotics (ISAPP) to revitalize the definition of a prebiotic as ‘a substrate that is selectively utilized by host micro-organisms conferring a health benefit’ [13]. Herein, we sought to verify and re-define prebiotic effects of the widely used soluble fiber, partially hydrolyzed guar gum (PHGG), applying state-of-the-art methodology in a controlled cohort trial named PAGODA.

In day-to-day clinical practice, prebiotics have been shown to exert beneficial effects in different clinical contexts such as gastrointestinal, cardiometabolic, mental health and bone diseases [40]. Accordingly, PHGG clinical studies have also demonstrated beneficial effects particularly in functional gastrointestinal disorders such as irritable bowel syndrome or constipation [41,42]. The specific study design of the PAGODA trial, involving three serial study periods, allowed measuring specific clinical effects in a time- and exposure-resolved manner. Confirmatory of previously published data [17,18], in our study, PHGG exposure was associated with an increase in stool frequency and a reduction in stool consistency according to the BSS score.

The prebiotic definition by the ISAPP group refers to the feature of ‘selective utilization’, which translates into utilization of the compound by specific members of the microbiota [13,43], thus conferring a fitness advantage with a respective impact on microbiota composition and function. Due technical limitations in earlier days, this was often equated with an expansion of Bifidobacteria and Lactobacilli and an increase in the abundance of SCFAs [13]. These prebiotic characteristics have also been demonstrated for PHGG [44]. In order to consider this definition, we have chosen a study design in which each study participant can serve his or her own control allowing to compensate for effects driven by the individual microbiota. To minimize the risk of a selection bias determined by the choice of the 16S chemistry, all samples were amplified and sequenced using both V1–V3 and V3–V4 primer pairs. To obtain functional insights we performed untargeted NMR spectroscopy in order to identify PHGG-induced metabolic pathways and to control for PHGG intake and its breakdown products.

Indeed, we identified PHGG-induced alterations of microbial communities with changes in the indices of species evenness and with an increase in pairwise intra-individual Bray–Curtis dissimilarities. Notably, the observed changes were largely reproducible between the two studied regions of the bacterial 16S gene. The nuanced differences between the findings from the two regions reflect their varying sensitivity for detection of certain bacterial groups. In accordance with the suggestions by Bindels et al. [45], we demonstrated that PHGG exerts broader effects on microbial ecology affecting 28 different taxa. In line with Bindels et al., our data provide further evidence that ‘selective utilization’ may be less dependent on specific taxa but on shared metabolic traits. Such metabolic traits may then provide secondary product that affect other groups microbes by cross-feeding highlighting the complexity of microbial ecology. Interestingly, the majority of these effects were transient and reversed during the washout period. As clinical effects and benefits along the prebiotic–microbiota–host axis are mechanistically linked by bacterial molecules arising from an altered microbial metabolic activity [39], we sought to identify changes in bacterial metabolites induced by PHGG. As expected and in line with previous research [16], induction of SCFAs was the most prominent effect, although additional functional changes in amino acid metabolism were identified. NMR spectroscopy made it possible to quantify fecal concentrations of the prebiotic PHGG itself along with its breakdown products. Interestingly, a daily intake of 15 g PHGG was fully metabolized by microbial metabolism and PHGG remained undetectable in the feces, whereas its cleavage products galactose and mannose were enriched. To link compositional data from 16S metagenomics with the functional metabolomic results, we employed interaction network analysis, which showed associations between OTU abundance and metabolites, particularly SCFAs, hinting at an accumulation of enzymatic functions of these bacterial species.

Another particularly interesting finding of this study was a strong inter-individual difference in response to PHGG between study participants. These differences were very pronounced with regard to clinical effects and generally stronger in male than in female participants. Nutritional data analyses revealed that women had a significantly higher baseline fiber intake, which could be a possible explanation for this discrepancy. However, differences in clinical responsiveness were not paralleled on the metagenomic level. This is consistent with findings reported by De Palma et al., who performed gnotobiotic models in animals that were conventionalized with either IBS or control stool. The authors did not find differences in the taxonomic composition between IBS- and control-conventionalized animals [46]. Nevertheless, IBS-D but not control feces induced typical IBS alterations in gut function and behavior, underscoring the importance of bacterial metabolites mechanistically linking the microbiota with human disease [46]. Strikingly, in our study, these gender-specific differences were reproducible on the metabolomic level, suggesting a mechanistic link between microbial function and clinical phenotype in our study. The absence of a gender difference in the effects of PHGG on a metagenomic level most likely reflects the functional convergence of individua, microbiotas, i.e., analogous biochemical functions are performed by different bacterial taxa within individual hosts.

Taken together, the highly individual responses to PHGG in our study further indicate the need for parameters enabling personalized prebiotic interventions. The complex relationships between the host, its commensal microbiota and their response to a specific prebiotic are influenced by multiple variables and makes a one-fits-all prebiotic becoming available unlikely [47]. Using our dataset, we were able to identify a steady state microbial configuration in combination with a low butyrate concentration to predict clinical response, i.e., an above-average increase in stool frequency and reduction in stool consistency, to PHGG.

## Supplementary Materials

The following are available online at https://www.mdpi.com/2072-6643/12/5/1257/s1, Table S1: Primer Sequences, Table S2: Baseline laboratory test results and medical data, Table S3: Intestinal well-being and symptoms during this study, Table S4: Comorbidities in study participants, Table S5: Lifestyle factors in study participants, Table S6: Shift values of reference peaks used for manual assignment of peak identities in NMR data, Figure S1: Baseline eating habits by gender, Figure S2: Results of V1–V3 region 16S sequencing, Figure S3: Individual trajectories of microbiota reconstitution V3–V4, Figure S4: Individual trajectories of microbiota reconstitution V1–V3, Figure S5: Differentially abundant OTUs in the V1–V3 dataset and their mapping on the genus level, and Figure S6: Targeted analysis of NMR spectra for levels of PHGG and its associated metabolites galactose and mannose.
