# Supplementary material for: Prebiotic Effects of Partially Hydrolyzed Guar Gum on the Composition and Function of the Human Microbiota—Results from the PAGODA Trial
Source: Nutrients. 2020 Apr 28;12(5):1257. doi: 10.3390/nu12051257 (PMC7281958; doi:10.3390/nu12051257)
Supplement: Supplementary file 1 [file nutrients-12-01257-s001.pdf]

Article

Prebiotic Effects of PHGG on the Composition and Function of the Human Microbiome – Results from the PAGODA Trial

Simon J Reider [1,2], Simon Moosmang [3], Judith Tragust [1], Lovro Trgovec-Greif [4], Simon Tragust [5], Lorenz Perschy [4], Nicole Przysiecki [1,2], Sonja Sturm [3], Herbert Tilg [2], Hermann Stuppner [3], Thomas Rattei [4], and Alexander R Moschen [1,2,*]

[1] Christian Doppler Laboratory for Mucosal Immunology, Medical University Innsbruck, Innsbruck, Austria
[2] Department of Internal Medicine I, Gastroenterology, Hepatology, Endocrinology & Metabolism, Medical University Innsbruck , Innsbruck , Austria
[3] Institute of Pharmacy/Pharmacognosy & Center for Molecular Biosciences Innsbruck, Leopold-Franzens Universität, Innsbruck, Austria
[4] Division of Computational Systems Biology, Department of Microbiology, University of Vienna, Vienna, Austria
[5] General Zoology / Institute of Biology, University Halle, Halle/Saale, Germany.

* Correspondence: alexander.moschen@i-med.ac.at

Supplementary Information

Table S1. Primer sequences

Primer	Primer sequence (5' -> 3')	Primer length (bp)	Amplicon size	Region
8F-YM	AGAGTTTGATYMTGGCTCAG	20	510 bp	V1-V3
517R	ATTACCGCGGCTGCTGG	17		
341F	CCTACGGGNGGCWGCAG	17	464 bp	V3-V4
805R	GACTACHVGGGTATCTAATCC	21		

Table S2. Baseline laboratory test results and medical data

	Sex	Mean	Std.Dev.	Minimum	Maximum
**Age**	male	30.38	7.42	23	44
	female	26	6.88	20	46
**BMI [kg/m^2]**	male	23.56	2.13	21.22	27.17
	female	20.4	1.86	17.78	23.45
**WHR**	male	0.79	0.06	0.7	0.84
	female	0.73	0.07	0.57	0.84
**WBC [G/L]**	male	5.03	0.82	4.1	6.3
	female	5.13	0.78	4	6.7
**Platelets [G/l]**	male	230.5	42.53	184	312
	female	226.45	28.94	183	284
**Hemoglobin [g/L]**	male	154.12	7.18	143	167
	female	132.45	7.63	124	150
**CRP [µmol/L]**	male	0.05	0.08	0	0.19
	female	0.03	0.07	0	0.24
**Calprotectin [µg/g]**	male	13.91	16.11	0	39.6
	female	7.67	25.45	0	84.4

BMI = Body Mass Index, WHR = Waist Hip Ratio; WBC = White blood cell count; CRP = C-reactive protein; Std.Dev. = Standard deviation

Table S3. Intestinal wellbeing and symptoms during the study

	Sex	Baseline (Wk 1-3)	Wk 4	Wk 5	Wk 6	Wk 7	Wk8	Wk9
Appetite Decrease	male	1 (+/- 0)	1 (+/- 0)	1 (+/- 0)	1 (+/- 0)	1 (+/- 0)	1 (+/- 0)	1 (+/- 0)
	female	1.18 (+/- 0.53)	1.18 (+/- 0.4)	1.18 (+/- 0.4)	1.09 (+/- 0.3)	1.18 (+/- 0.6)	1.18 (+/- 0.6)	1.09 (+/- 0.3)
Appetite Increase	male	1 (+/- 0)	1 (+/- 0)	1 (+/- 0)	1.12 (+/- 0.35)	1.12 (+/- 0.35)	1.38 (+/- 0.74)	1.25 (+/- 0.46)
	female	1.24 (+/- 0.61)	1.18 (+/- 0.6)	1.27 (+/- 0.47)	1.36 (+/- 0.67)	1.09 (+/- 0.3)	1.09 (+/- 0.3)	1.18 (+/- 0.6)
Bristol Stool Scale	male	3.27 (+/- 0.97)	3.7 (+/- 0.59)	4.11 (+/- 0.77)	3.92 (+/- 1.26)	3.6 (+/- 1.15)	3.54 (+/- 0.69)	3.27 (+/- 0.79)
	female	3.64 (+/- 1.08)	3.71 (+/- 0.91)	3.54 (+/- 0.71)	3.52 (+/- 0.81)	3.72 (+/- 0.77)	3.33 (+/- 0.88)	3.4 (+/- 0.78)
Stool Frequency [n/day]	male	1.11 (+/- 0.37)	1.33 (+/- 0.54)	1.34 (+/- 0.56)	1.48 (+/- 0.61)	1.32 (+/- 0.48)	1.21 (+/- 0.46)	1.23 (+/- 0.45)
	female	1.15 (+/- 0.53)	1.17 (+/- 0.53)	1.14 (+/- 0.34)	1.24 (+/- 0.35)	1.23 (+/- 0.4)	1.12 (+/- 0.37)	1.19 (+/- 0.42)
Diarrhoea	male	1.38 (+/- 0.77)	1.38 (+/- 0.52)	1.38 (+/- 0.74)	1.5 (+/- 0.93)	1.25 (+/- 0.71)	1.12 (+/- 0.35)	1.12 (+/- 0.35)
	female	1.3 (+/- 0.64)	1.18 (+/- 0.4)	1.18 (+/- 0.4)	1.18 (+/- 0.4)	1.18 (+/- 0.4)	1 (+/- 0)	1.36 (+/- 0.81)
Flatulation	male	1.71 (+/- 0.69)	2.12 (+/- 1.13)	2.75 (+/- 0.71)	2.38 (+/- 1.19)	1.88 (+/- 0.83)	1.88 (+/- 0.99)	1.75 (+/- 0.89)
	female	1.82 (+/- 0.73)	2.55 (+/- 1.04)	2.55 (+/- 0.82)	2.27 (+/- 1.1)	1.45 (+/- 0.69)	1.36 (+/- 0.5)	1.27 (+/- 0.47)

Nausea	male	1.21 (+/- 0.66)	1.25 (+/- 0.71)	1.5 (+/- 0.76)	1.25 (+/- 0.46)	1.25 (+/- 0.46)	1.25 (+/- 0.46)	1.25 (+/- 0.46)
	female	1.06 (+/- 0.24)	1.09 (+/- 0.3)	1 (+/- 0)	1.09 (+/- 0.3)	1 (+/- 0)	1 (+/- 0)	1.09 (+/- 0.3)
Obstipation	male	1.04 (+/- 0.2)	1.12 (+/- 0.35)	1.25 (+/- 0.46)	1.12 (+/- 0.35)	1.25 (+/- 0.71)	1.25 (+/- 0.71)	1.12 (+/- 0.35)
	female	1.15 (+/- 0.36)	1.18 (+/- 0.4)	1.18 (+/- 0.4)	1.27 (+/- 0.65)	1.09 (+/- 0.3)	1.36 (+/- 0.5)	1.27 (+/- 0.65)
Pain	male	1.33 (+/- 0.7)	1.5 (+/- 0.53)	1.5 (+/- 0.76)	1.38 (+/- 0.74)	1.25 (+/- 0.46)	1.12 (+/- 0.35)	1.25 (+/- 0.46)
	female	1.42 (+/- 0.66)	1.36 (+/- 0.67)	1.64 (+/- 0.81)	1.45 (+/- 0.82)	1 (+/- 0)	1.36 (+/- 0.67)	1.09 (+/- 0.3)
Quality of life	male	6.58 (+/- 0.5)	6.12 (+/- 0.64)	6.25 (+/- 0.89)	6.75 (+/- 0.46)	6.75 (+/- 0.46)	6.5 (+/- 0.76)	6.5 (+/- 0.53)
	female	6.18 (+/- 0.88)	6.27 (+/- 0.79)	6.09 (+/- 0.83)	6.45 (+/- 0.82)	6.45 (+/- 0.69)	6.73 (+/- 0.65)	6.64 (+/- 0.5)

Symptoms were quantified using a questionnaire with a graduation (possible answers) of never (1), occasionally (2) or regularly (3). Values in this table represent mean and standard deviation. Stool frequency was quantified in number per day and quality of life was ranked from 0 to 9 (with 9 being the highest quality possible).

Table S4. Comorbidities in study participants

	female	male
Hypothyreosis	3	0
Asthma	1	0
Migraine	4	0
Lactose intolerance	0	1
History of appendectomy	1	0
Pollen allergy	4	2
Nut allergy	1	0

Number of participants suffering from the listed comorbidities. Other than these conditions, participants were healthy.

Table S5. Lifestyle Factors in study participants

	Never	Occasionally	Regularly
Smoking	18 (94.7 %)	0 (0 %)	1 (5.3 %)
Alcohol	2 (20.5 %)	16 (84.2 %)	1 (5.3 %)
Sports	1 (5.3 %)	7 (36.8 %)	11 (57.9 %)

Table S6. Shift values of reference peaks used for manual assignment of peak identities in NMR data

Metabolite name	Shift Value
Acetate	1.9241
Propionate CH3	1.047
Propionate CH3	1.0598
Propionate CH3	1.0725
Propionate CH2	2.1673
Propionate CH2	2.1801
Propionate CH2	2.1929
Propionate CH2	2.2057
Butyrate CH3	0.88616
Butyrate CH3	0.89861
Butyrate CH3	0.91106
Butyrate CH2b	1.5311
Butyrate CH2b	1.5432
Butyrate CH2b	1.5557
Butyrate CH2b	1.5678
Butyrate CH2b	1.5802
Butyrate CH2b	1.5927
Butyrate CH2a	2.1495
Butyrate CH2a	2.1616
Butyrate CH2a	2.174
Galactose	5.2706
Galactose	5.2729
Mannose	5.1942
Mannose	5.1915
PHGG	5.04

Supplementary Figure 1

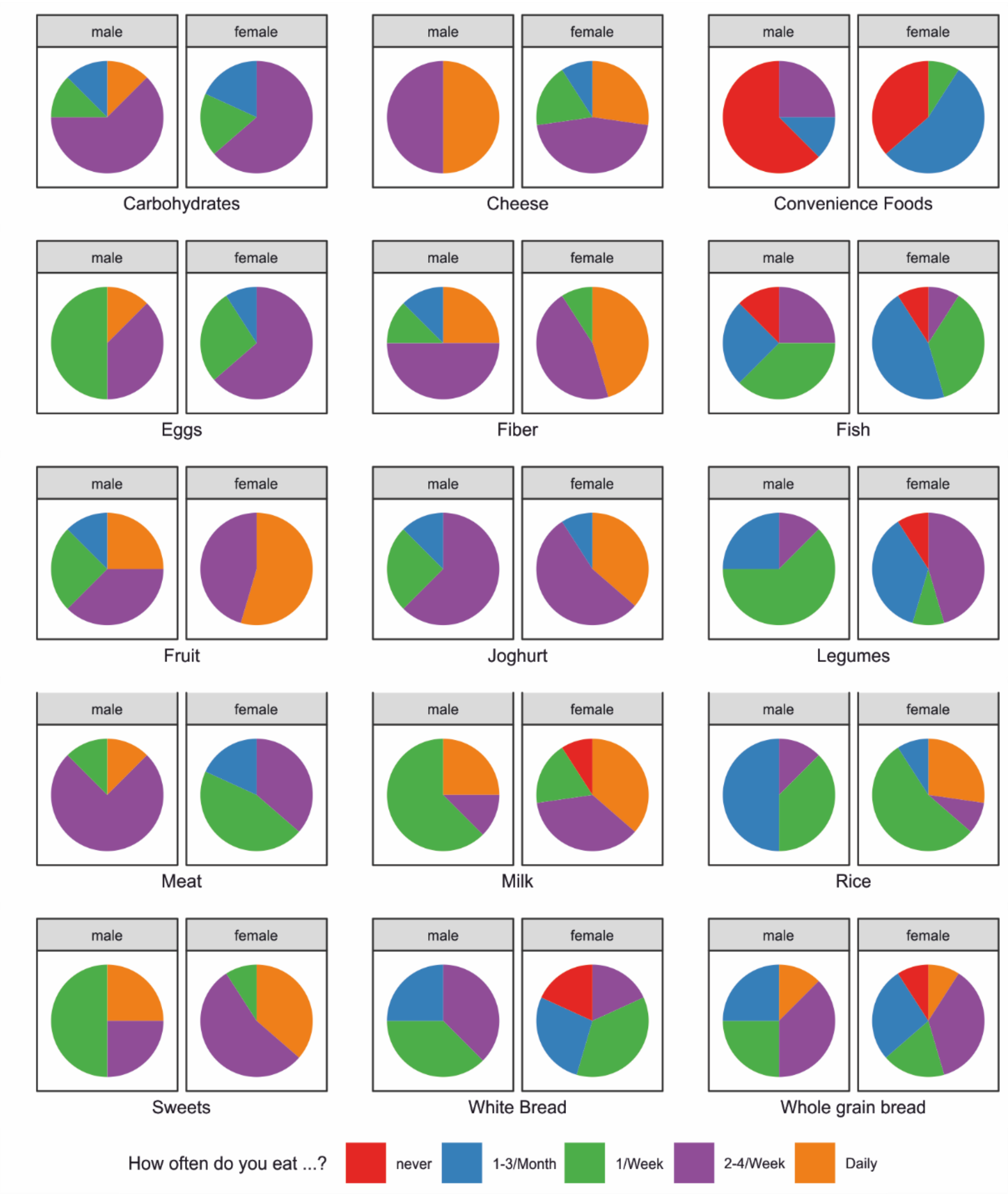

Figure S1. Baseline eating habits by gender. Female participants reported a higher consumption of fiber and fruit and less intake of red meat.

Supplementary Figure 2

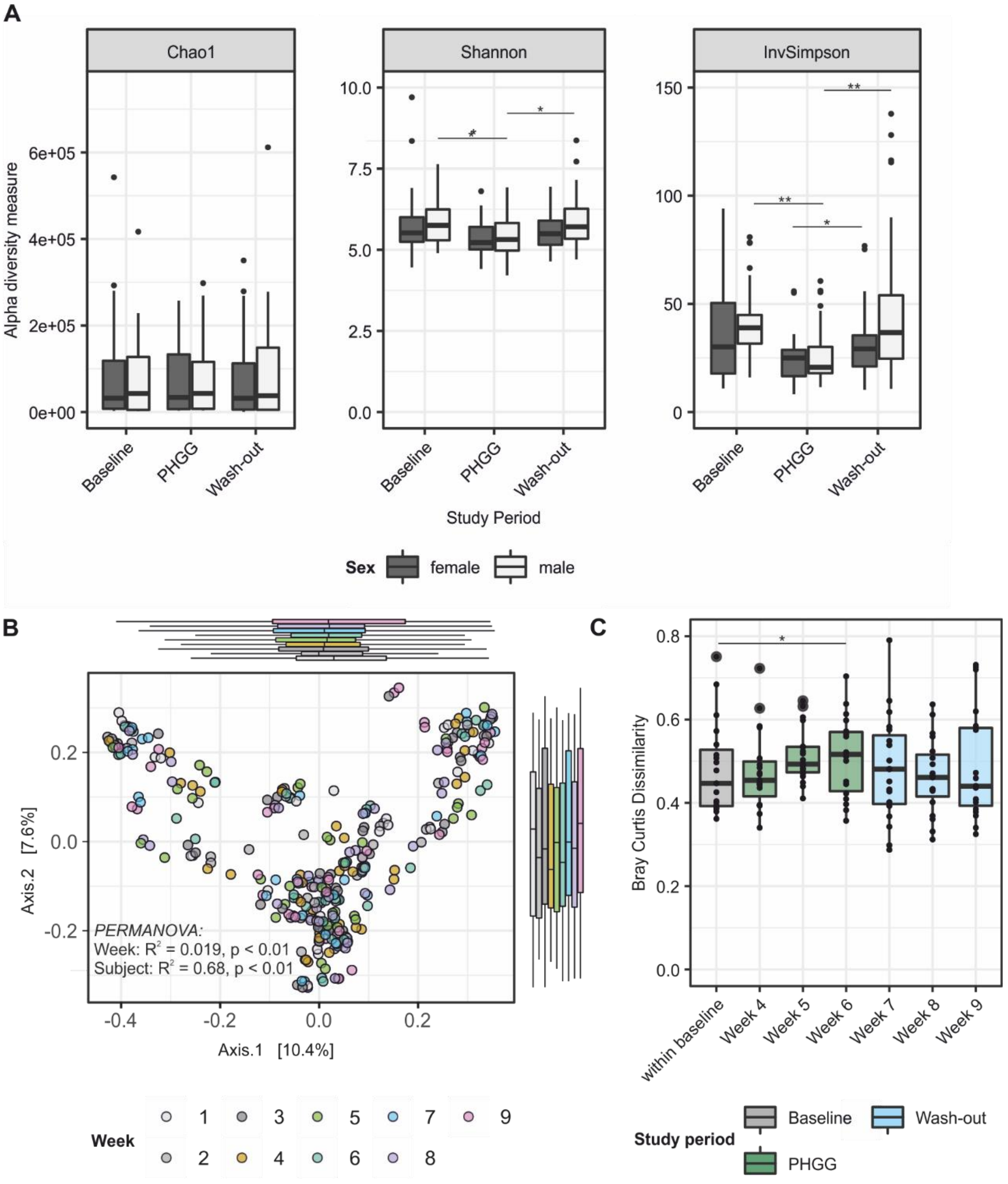

Figure S2. Results of V1-V3 region 16S sequencing corresponding to Figure 2. **(A)** α-diversity decreases during intervention in both sexes but returns to baseline values during the washout period (pairwise Wilcoxon test); **(B)** In a principle component analysis of microbial composition over time, study week (i.e. PHGG supplementation status) was identified as a significant determinant (PERMANOVA permutational analysis of variance); **(C)** Pairwise analysis of Bray-Curtis dissimilarity over time: Community composition becomes significantly more dissimilar by week 6 than expected during steady state (Wilcoxon test). After the intervention period, the microbiota returns to its baseline state. PHGG = partially hydrolyzed guar gum; *$p < 0.05$, **$p < 0.01$.

Supplementary Figure 3

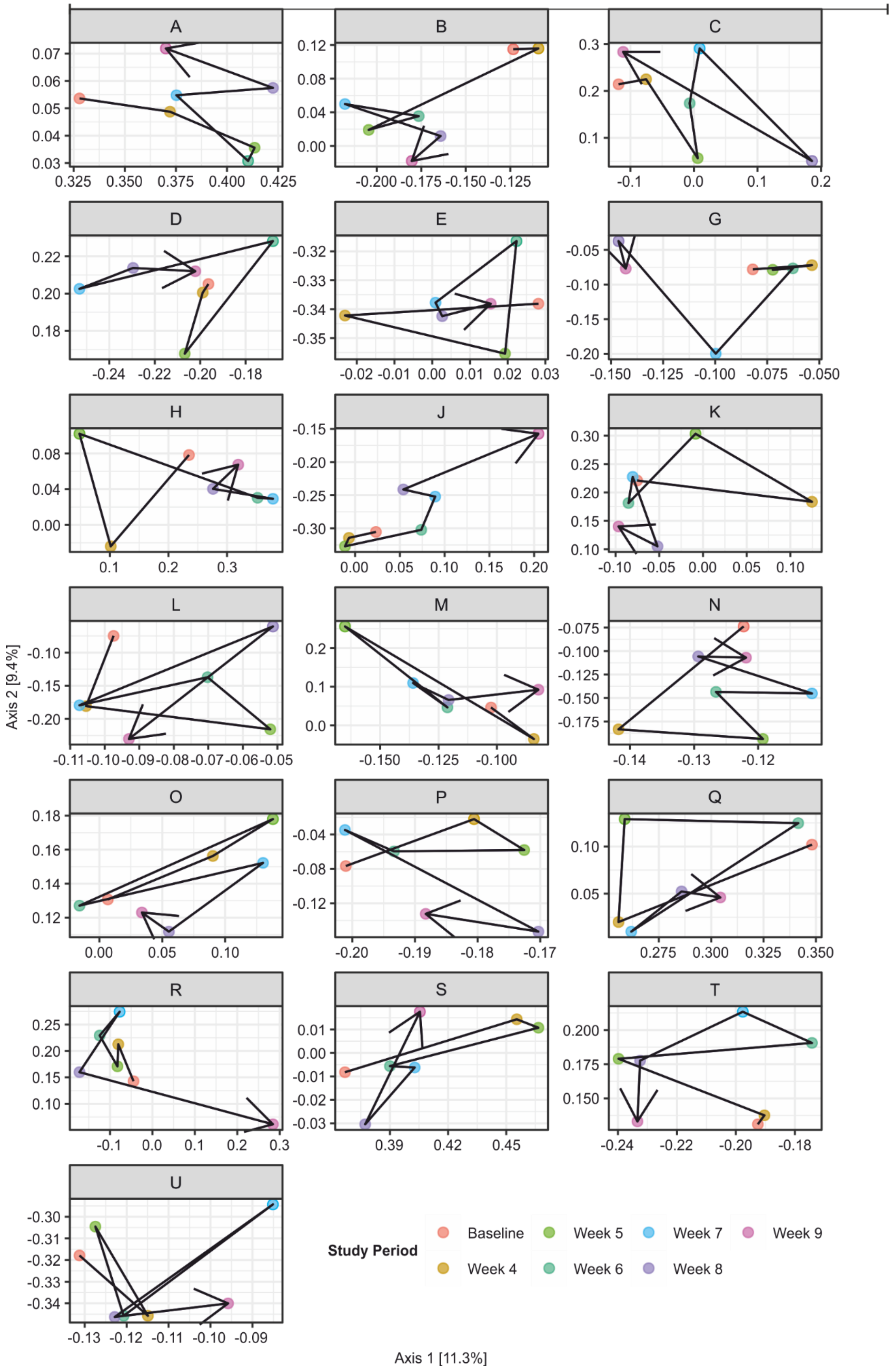

Figure S3. Individual trajectories of microbiome reconstitution. Primary coordinate analysis of Bray-Curtis dissimilarities over time; V3-V4 region.

Supplementary Figure 4

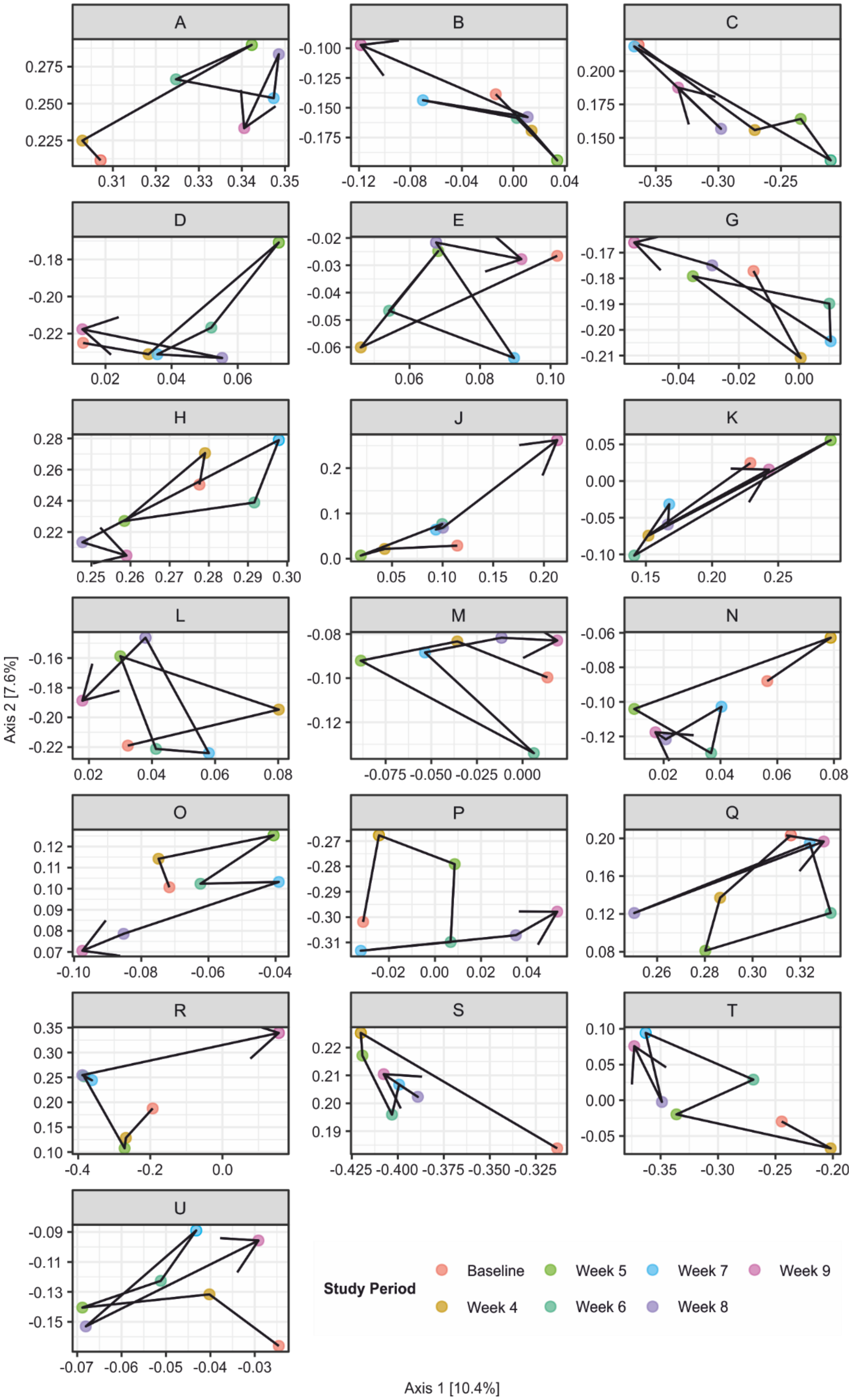

Figure S4. Individual trajectories of microbiome reconstitution. Primary coordinate analysis of Bray-Curtis dissimilarities over time; V3-V4 region.

Supplementary Figure 5

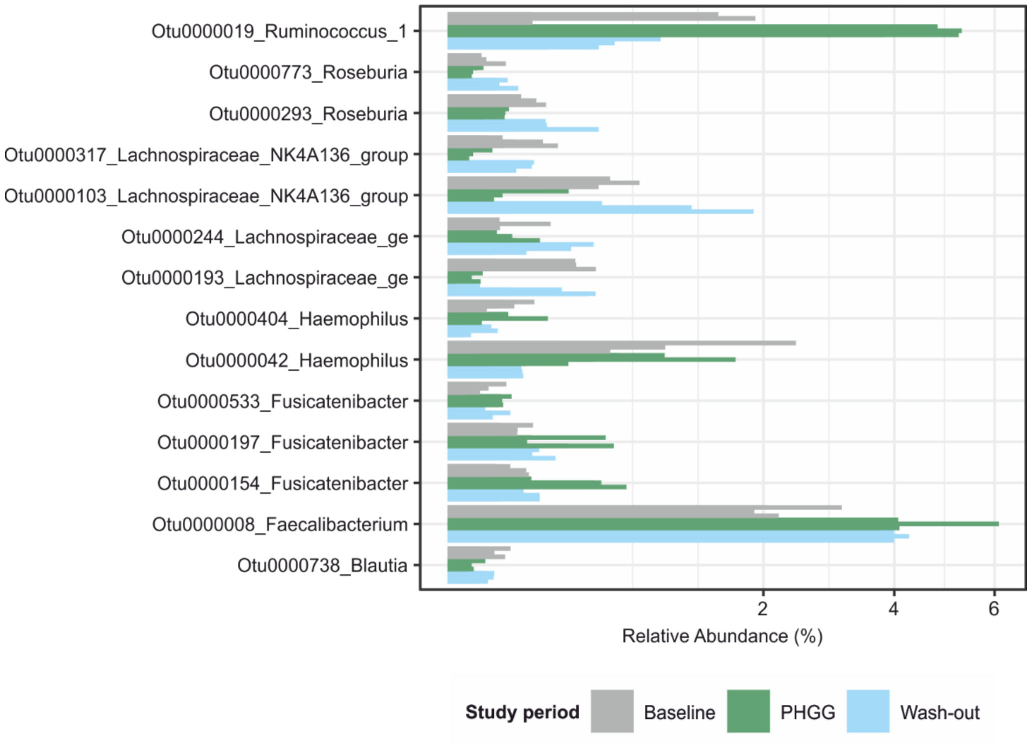

Figure S5. Differentially abundant OTUs in the V1-V3 dataset and their mapping on the genus level. Testing was done using a negative binomial model implemented in DESeq2 and a cutoff of 0.01 for the adjusted p-value. OTU = operational taxonomic unit; UCG = unknown classification group, PHGG = partially hydrolyzed guar gum.

Supplementary Figure 6

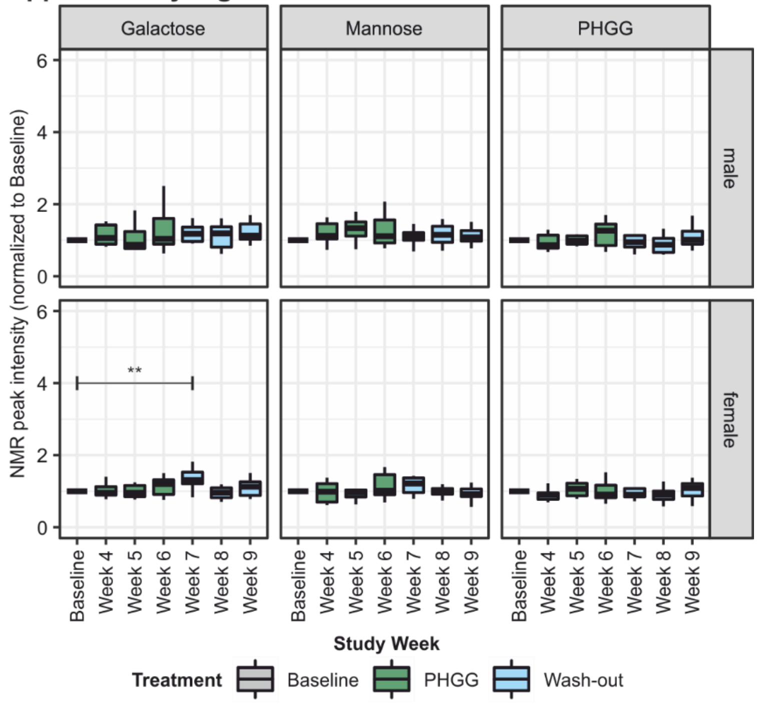

Figure S6. Targeted analysis of NMR spectra for levels of PHGG and its associated metabolites galactose and mannose. Values from the first 3 weeks of the study were averaged („Baseline“). (PHGG = partially hydrolyzed guar gum; Statistics: pairwise Wilcoxon tests between baseline and week 4 – week 8; Bonferroni-corrected; $*p < 0.05$, $**p < 0.01$).
